# High-Carbohydrate Diet Alleviates the Oxidative Stress, Inflammation and Apoptosis of *Megalobrama amblycephala* Following Dietary Exposure to Silver Nanoparticles

**DOI:** 10.3390/antiox10091343

**Published:** 2021-08-25

**Authors:** Fang Chen, Cai-Yuan Zhao, Jun-Feng Guan, Xiao-Cheng Liu, Xiang-Fei Li, Di-Zhi Xie, Chao Xu

**Affiliations:** 1 College of Marine Sciences, South China Agricultural University, No.483 Wushan Road, Guangzhou 510642, China; Fangchen@scau.edu.cn (F.C.); a374168380@scau.edu.cn (J.-F.G.); lxiao0805@scau.edu.cn (X.-C.L.); xiedizhi@scau.edu.cn (D.-Z.X.); 2 State Key Laboratory of Biocontrol, Southern Marine Sciences and Engineering Guangdong Laboratory (Zhuhai), School of Marine Sciences, Sun Yat-Sen University, Guangzhou 510275, China; zhaocy5@mail.sysu.edu.cn; 3 Key Laboratory of Aquatic Nutrition and Feed Science of Jiangsu Province, College of Animal Science and Technology, Nanjing Agricultural University, No.1 Weigang Road, Nanjing 210095, China; xfli@njau.edu.cn

**Keywords:** silver nanoparticles, apoptosis, inflammation, daily component, *Megalobrama amblycephala*

## Abstract

A 12-week feeding trial was performed to evaluate the effects of high-carbohydrate diet on oxidative stress, inflammation and apoptosis induced by silver nanoparticles (Ag-NPs) in *M*. *amblycephala*. Fish (20.12 ± 0.85 g) were randomly fed four diets (one control diet (C, 30% carbohydrate), one control diet supplemented with 100 mg kg^−1^ Ag-NPs (CS), one high-carbohydrate diet (HC, 45% carbohydrate) and one HC diet supplemented with 100 mg kg^−1^ Ag-NPs (HCS)). The results indicated that weight gain rate (WGR), specific growth rate (SGR), antioxidant enzyme (SOD and CAT) activities and expression of Trx, Cu/Zn-SOD, Mn-SOD, CAT and GPx1 of fish fed CS diet were all remarkably lower than those of other groups, whereas the opposite was true for plasma IL 1β and IL 6 levels, liver ROS contents, hepatocytes apoptotic rate, AMP/ATP ratio, AMPKα, P 53 and caspase 3 protein contents and mRNA levels of AMPKα 1, AMPKα 2, TXNIP, NF-κB, TNFα, IL 1β, IL 6, P 53, Bax and caspase 3. However, high-carbohydrate diet remarkably increased WGR, SGR, liver SOD and CAT activities, AMPKα protein content and mRNA levels of antioxidant genes (Cu/Zn-SOD, Mn-SOD, CAT and GPx1), anti-inflammatory cytokines (IL 10) and anti-apoptotic genes (Bcl 2) of fish facing Ag-NPs compared with the CS group, while the opposite was true for liver ROS contents, hepatocytes apoptotic rate, P 53 and caspase 3 protein contents, as well as mRNA levels of TXNIP, NF-κB, TNFα, IL 1β, IL 6, P 53, Bax and caspase 3. Overall, high-carbohydrate diet could attenuate Ag-NPs-induced hepatic oxidative stress, inflammation and apoptosis of *M*. *amblycephala* through AMPK activation.

## 1. Introduction

At present, silver nanoparticles (Ag-NPs) are regarded as the most commercialized nanomaterial worldwide. Due to their strong antimicrobial properties, Ag-NPs have been commonly applied in many consumer products including plastics, metals, textiles, cosmetics and cleaning products, as well as medical and veterinary devices [1]. However, increasing applications of Ag-NPs have led to their enhanced accumulation in aquatic environment through water run-off. Once in the aquatic environment, the residues of Ag-NPs can cause several adverse effects, thereby threatening fish health. Reports on the effects of Ag-Nps have shown that they cause oxidative stress and inflammation of tissues in medaka (*Oryzias latipes*) following 2-week exposure [2], and they have been shown to lead to a reduction in membrane integrity and cellular metabolic activity in rainbow trout (*Onchorhyncus mykiss)* [3]. In addition, high mortality rate and growth retardation were also observed in zebrafish (*Danio rerio*) who encountered high concentrations of Ag-Nps [4]. Considering culturing profit, it is necessary to find effective approaches to alleviate the side effects induced by Ag-Nps in fish.

An accumulating body of evidence suggests that Ag-Nps have the ability to interact with biological tissues and generate reactive oxygen species (ROS) that are considered to be a potential mechanism of toxicity [1]. In fact, ROS over-production can induce the generation of multiple pro-inflammatory cytokines (such as tumor necrosis factor α (TNF α), interleukin 1β (IL 1β) and interleukin 6 (IL 6)) by activating some major pro-inflammatory transcription factors, such as nuclear factor kappa B (NF-κB), thereby resulting in inflammation [5,6,7,8]. Meanwhile, pro-inflammatory cytokines, such as TNF α and IL 6, can trigger apoptosis by up-regulating transcriptions of pro-apoptotic genes, such as Bcl 2 family member Bax, resulting in programmed cell death [9,10]. Regarding these processes, AMP-activated protein kinase (AMPK) has attracted considerable attention due to its potent effects on the regulation of intracellular oxidative stress, as well as its ability to protect cells from damage [11]. Generally, activated AMPK can regulate multiple biological processes, including oxidative responses, inflammation and apoptosis, such as (a) a decrease in reactive oxygen species (ROS) production by accelerating thioredoxin-interacting protein (TXNIP) degradation [12]; (b) suppression of the NF-κB pro-inflammatory pathway [13]; and (c) inhibition of the P 53 pro-apoptotic pathway [14]. However, the above findings were mainly derived from mammals. At present, evidence has indicated that fish exposed to Ag-NPs could cause acute oxidative stress accompanied by high concentrations of ROS [15,16]. In addition, the high mRNA levels of pro-inflammatory cytokines in tissues were also observed during Ag-NPs exposure [17]. However, the relevant physiological basis in fish is still barely understood, and it warrants further studies.

Dietary factors play pivotal roles in modulating the health of fish. As a macronutrient, carbohydrates can provide energy for the normal growth, development and health maintenance of fish. In addition, toxicological studies have demonstrated that dietary starch could reduce the toxicity of chemical drugs, including oxytetracycline, in organisms by changing the conjugation of drug and metabolites formation [18]. These findings highlight that, in the evaluation of chemical toxicity, dietary carbohydrates play important roles in the modulation of toxic effects. However, currently, no study has explored the potential use of dietary carbohydrates in modulating metal nanoparticles-induced side effects in fish. In view of this, we adopted blunt snout bream (*Megalobrama amblycephala*) (an economically important fish cultured in China) as an experimental animal to explore the effects of dietary carbohydrate levels on the growth, oxidative stress, inflammation and apoptosis of fish following dietary exposure to Ag-NPs. To the best of our knowledge, this is the first study to investigate the influence of dietary carbohydrate on adverse effects of metal nanoparticles in fish. The findings obtained here will benefit our understanding of the physiological mechanisms used by dietary carbohydrates to modulate fish facing Ag-NPs-induced side effects, and they are therefore helpful in relation to the healthy development of the aquaculture industry.

## 2. Materials and Methods

### 2.1. Ethics Statement

All procedures were performed in accordance with the National Institute of Health guide for the care and use of laboratory animals (NIH Publications No. 8023, revised 1978) and approved by the Institutional Animal Care and Use Committee of South China Agricultural University.

### 2.2. Silver Nanoparticles Preparation

Silver nanopowder (cat. no 576832) with purity of 99.5% was supplied by Sigma Aldrich Company (Cambridge, UK), and its particle size was <100 nm. Ag-NPs of 50 mg L^−1^ were dispersed by sonication in Milli-Q water for 1 h (sonicator, 250 W, 40 kHz, 30 °C; Elma TI-H-20, Bandelin, Berlin, Germany). After sonication, all buffers were further filtered by a 0.2 μm nylon membrane filter. The distribution and size of Ag-NPs were determined by transmission electron microscopy (TEM) using a JEOL JEM-1220 EX microscope (JEOL Ltd., Tokyo, Japan). Zeta potential of Ag-NPs was analyzed by a Zetasizer Nano-ZS90 (Malvern, Worcestershire, UK) using an electrophoretic light-scattering method. All measurements were taken in triplicate.

### 2.3. The Experimental Diets and Feeding Trial

First, two experimental diets were formulated to contain two dietary carbohydrate (namely nitrogen-free extract) levels: 30% and 45% (Table 1). According to our previous studies, the optimal dietary carbohydrate level for juvenile *M. amblycephala* is 29–32% [19]. Therefore, a diet containing 30% carbohydrate was adopted as the control, while a diet of 45% was designated as the high-carbohydrate (HC) diet. Then, some food pellets were supplemented with a stock dispersion of silver nanopowder, according to the method by Clark et al. (2019) [20]. Briefly, the dosing dispersions for mixing with the diets were prepared by sonicating (UC-5000 ultrasonic bath, Langee, Shenzhen, China) 100 mL of a nominal stock concentration of 1 g L^−1^ of Ag-NPs prepared in ultrapure water for 1 h. Nanoparticle tracking analysis (NTA) was carried out to confirm Ag-NPs material could be dispersed adequately in these stocks. The mean (±SEM) hydrodynamic diameter was 24 ± 2 nm for 1 mg L^−1^ Ag-NPs. This was slowly added to 900 g of the diet, and then gently mixed with a food mixer (1101049, Xiaomi, Foshan, China). A solution of 10 g of porcine gelatine (>98% purity, Sigma, Billerica, MA, USA) in 100 mL of ultrapure water was prepared by gentle heating to 40 °C, allowed to cool for 20 min, and then gently poured over the diet and mixed in for 20 min. The unexposed diet was prepared in exactly the same way but dosed with ultrapure water without Ag-NPs. All diets were dried in a ventilated oven at 30 °C and stored at −20 °C in plastic-lined bags until use. Herein, four experimental diets (namely C diet (30% carbohydrate), CS diet (C diet supplemented with 100 mg kg^−1^ Ag-NPs), HC diet (45% carbohydrate) and HCS diet (HC diet supplemented with 100 mg kg^−1^ Ag-NPs) were produced for a 12-week feeding trial. The actual Ag-NPs contents of the experimental diets were measured using the method of inductively coupled plasma mass spectrometry.

Juvenile *M. amblycephala* were bought from a local fish hatchery (Yangzhou, China). After acclimating to experimental facilities, a total of 320 *M. amblycephala* (initial weight 20.12 ± 0.85 g) was allocated to 16 indoor tanks (300 L volume) at a rate of 20 fish per tank. Fish in each tank were randomly assigned to one of four experimental diets. Each diet was tested in four tanks. Daily feeding time was 08:30, 12:30 and 17:30. Water temperature varied from 27 to 29 °C; dissolved oxygen was maintained above 5.0 mg L^−1^; pH ranged from 7.3 to 7.6; total ammonia nitrogen and nitrite were kept <0.4 and 0.01 mg L^−1^, respectively; and the photoperiod was 12 h:12 h (dark:light).

### 2.4. Sample Collection

After 12 weeks, following a 24 h fast, all the fish in each tank were counted and weighed. In total, four fish per tank were immediately euthanized by MS-222 at 100 mg L^−1^. Blood was rapidly sampled from the caudal vein, centrifuged (3000× *g*, 10 min, 4 °C) and kept at −80 °C until analysis. Then, liver samples from four fish per tank were collected, and then fixed in 4% paraformaldehyde for histological analysis. In addition, liver from another four fish per tank was sampled, and then snap frozen in liquid nitrogen and stored at −80 °C.

### 2.5. Analysis of Proximate Composition and Plasma and Liver Biochemical Indices

Moisture, crude lipid, crude protein, ash, gross energy and crude fiber contents of diets were assayed by AOAC (1990) [21]. Moisture was measured by an oven at 105 °C until at a constant weight; crude protein (nitrogen × 6.25) was determined by the micro-Kjeldahl method using an Auto Kjeldahl System (FOSS KT260, Zurich, Switzerland); crude lipid was determined by solvent extraction using a Soxtec System (Soxtec System HT6, Tecator, Höganäs, Sweden); ash was determined by combustion at 550 °C for 4 h; gross energy was determined using a bomb calorimeter (PARR 1281, Parr Instrument Company, Moline, IL, USA); nitrogen-free extract was determined by fritted glass crucible method using an automatic analyzer (ANKOM A2000i, New York, NY, USA).

Plasma activities of aspartate aminotransferase (AST) and alanine aminotransferase (ALT) were assayed according to Habte-Tsion et al. (2016) [22]. Plasma levels of interleukin 1β (IL 1β) and interleukin 6 (IL 6) were measured by cytokine-specific enzyme-linked immunosorbent assay (ELISA) kits according to the manufacturer’s instructions (R and D Systems, Minneapolis, MN, USA, no. MTA00B, D6050 and DCP00).

Liver ROS levels were determined by measuring the oxidative conversion of cell permeable 2′, 7′dichlorofluorescein diacetate (DCF-DA) [23]. Briefly, 20 μL of liver homogenate was pipetted into each well of a 96-well plate and allowed to warm to room temperature for 5 min. At that time, 100 μL physiological saline and 5 μL of 2,7-dichlorofluorescin diacetate (DCFH-DA, dissolved in DMSO, 10 μmoL/L final concentration) were added to every well and the plate was incubated at 37 °C for 30 min. The conversion of DCFH to the fluorescent product DCF was measured using a TECAN spectrofluorometer with excitation/emission at 485/530 nm (Tecan, Mannedorf, Switzerland). Background fluorescence (conversion of DCFH to DCF in the absence of homogenate) was corrected by the inclusion of parallel blanks.

The liver tissues were rinsed and homogenized in 50 mM ice-cold potassium phosphate buffer (1:8, *w*/*v*, pH 7.0). Then, the homogenate was centrifuged for 10 min at 10,000 rpm and 4 °C. The supernatant was used to measure the protein and malondialdehyde (MDA) contents, total antioxidant capacity (T-AOC), as well as total superoxide dismutase (SOD) and catalase (CAT) activities. Protein contents were measured by the method used by Bradford (1976) [24] using bovine serum albumin as a standard. Hepatic T-AOC, SOD and CAT were evaluated using commercial kits (Jiancheng Bioengineering Institute, Nanjing, China) according to the manufacturer’s instructions. MDA contents were detected according to Zhao et al. (2016) [25] with thiobarbituric acid. A mixture of 100 μL of homogenate, 0.37% SDS, 6.8% acetic acid (pH 3.5) and 1% TBA was incubated at 80–90 °C for 1 h and then centrifuged at 3000× *g* for 15 min. The absorbance at 532 nm was measured when the mixture approached room temperature.

### 2.6. Analysis of Liver Histology and ATP and AMP Contents

For the detection of apoptosis in the liver, TUNE (terminal-deoxynucleotidyl transferase mediated nick end labeling) staining was executed by an in situ cell death detection kit, POD (Roche Applied Science, Mannheim, Germany). Cells with blue nuclei were considered TUNEL-negative and counted as normal cells. The apoptosis rate (%) = 100% × the number of positive cells/total cells.

The liver from four fish per tank was homogenized in perchloric acid buffer and the homogenate was centrifuged at 10,000× *g* for 10 min. Then, the supernatant was separated, and neutralized with 0.5 volumes of 2 mol L^−1^ potassium hydroxide cocktail. Liver ATP contents were determined enzymatically using a Varian Cary UV/Vis spectrophotometer [26]. The generation of NADPH was in direct proportion to the amount of ATP in the extract, once glucose-6-phosphate was depleted. The assay conditions were: 100 mM triethanolamine HCl adjusted to pH 7.6 with NaOH, 4 mM MgCl_2_, 2 mM glucose, 2 mM NADP, 2.8 units mL^−1^ glucose-6-phosphate dehydrogenase and 1.8 units mL^−1^ hexokinase. Hexokinase was omitted initially to deplete glucose-6-phosphate in the extract. After the addition of hexokinase, the reaction was followed to completion at 340 nm to assess ATP contents. Liver AMP contents were measured following NADH utilization by lactate dehydrogenase (LDH) in proportion to pyruvate production by pyruvate kinase (PK) according to Adam (1965) [27]. The assay conditions were: 100 mM triethanolamine HCl adjusted to pH 7.6 with NaOH, 1 mM PEP, 33.4 mM MgSO_4_, 0.12 M KCl, 0.36 mM NADH, 24 units mL^−1^ lactate dehydrogenase, 18 units mL^−1^ PK. AMP contents were determined according to the difference in optical density before and after the addition of PK and myokinase.

### 2.7. Analysis of Western Blot (WB) and RT-PCR

WB analysis (20 μg of liver protein) was performed using anti-AMPKα (#2532, Cell Signaling Technology, Danvers, MA, USA), P 53 (A0263, ABclonal, Wuhan, China), caspase 3 (13847, Abcam, Cambridge, MA, USA) and anti-β-tubulin (10094-1-AP, Proteintech, Rosemont, IL, USA) antibodies. These antibodies have all been shown to successfully cross-react with *M. amblycephala* proteins. The signals of WB were quantitatively assayed by ImageJ 1.44 image analysis software.

Total RNA was extracted from the liver using TRIzol reagent (Invitrogen, Carlsbad, CA, USA), and its RNA concentration was detected by a NanoDrop spectrophotometer (NanoDrop Technologies, Wilmington, DE, USA). Then, cDNA was synthesized using PrimeScript Ist strand cDNA synthesis kit (Takara, Tokyo, Japan). Finally, the expression levels of target genes were detected by real-time PCR with specific primers (Table 2) under the SYBGREEN-based Light Cycle 96 system (Roche, LC96). EF1α (elongation factor 1 alpha) gene was used as an endogenous control because of the non-significant changes in the Ct value between the treatments. The expression levels of target genes were normalized by the expression of EF1α, and the relative expression levels were calculated by 2^−ΔΔCt^ method [28].

### 2.8. Statistical Analyses

Data were analyzed using one-way analysis of variance (ANOVA) by SPSS 22.0 statistical software. Tukey’s HSD multiple comparison test was adopted to rank the means. All data are presented as means ± S.E.M (standard error of the mean) of four replicates. Statistical significance was set at *p* < 0.05.

## 3. Results

### 3.1. Characterization of the Ag-NPs

**Figure 1:**
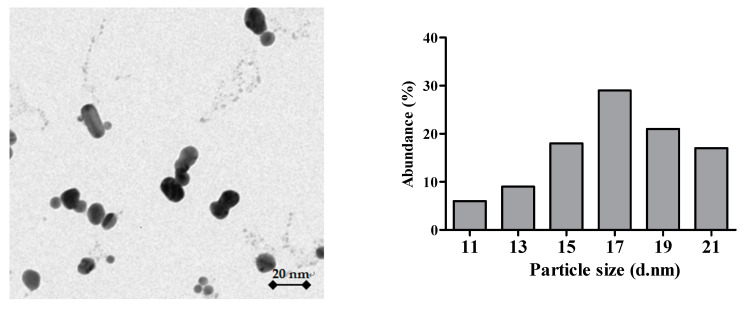
TEM image of AgNPs and its particle size distribution histogram.

The size of Ag-NPs measured with TEM ranged from 11 to 21 nm (average value 17.32 ± 4.09 nm) (Figure 1). The zeta potential for Ag-NPs was −55.6 ± 2.0 mV.

### 3.2. Growth Performance and Feed Utilization

Growth performance and feed utilization of blunt snout bream subjected to different treatments are shown in Table 3. No mortality was observed during the 12-week feeding trial. Initial weight, relative feed intake (RFI) and feed conversion ratio (FCR) showed no significant differences (*p* > 0.05) among all the treatments. Final weight, weight gain rate (WGR) and specific growth rate (SGR) of fish fed the CS diet were all lower than those of other groups. However, their values in the HCS group were significantly (*p* < 0.05) higher than those of the CS and HC groups. 

### 3.3. Plasma and Liver Biochemistry Parameters

As can be seen from Table 4, plasma alanine transaminase (AST), aspartate aminotransferase (ALT), interleukin 1β (IL 1β) and interleukin 6 (IL 6), as well as liver reactive oxygen species (ROS) and malondialdehyde (MDA) contents of the CS group, were all significantly (*p* < 0.05) higher than those of other groups, whereas the opposite was true for liver values of total anti-oxidation capacity (T-AOC), superoxide dismutase (SOD) and catalase (CAT). The values of AST, IL 6, ROS and MDA of the HCS group were significantly (*p* < 0.05) higher than those of the C group, whereas the opposite was true for T-AOC, SOD and CAT. In addition, the values of AST, IL 6, ROS, MDA, T-AOC, SOD and CAT showed no significant differences (*p* > 0.05) between the HC and HCS groups.

### 3.4. Liver Histological Analysis

**Figure 2:**
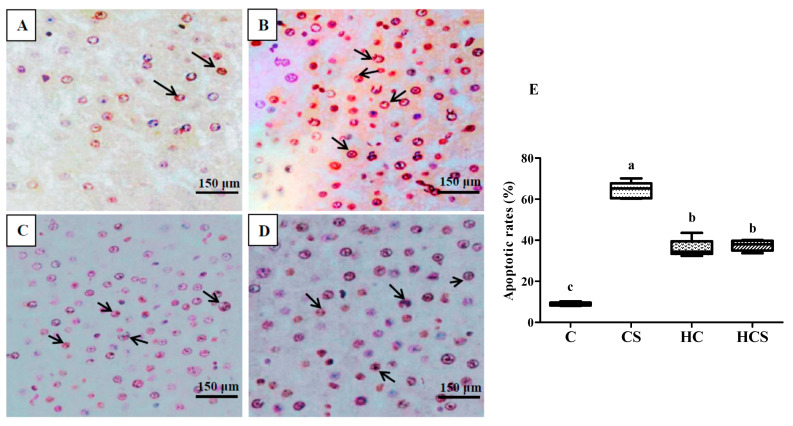
TUNEL detection of hepatic apoptosis of *M. amblycephala* subjected to different treatments: (**A**) the C group; (**B**) the CS group; (**C**) the HC group; (**D**) the HCS group. (**E**) **is apoptotic rates. The brown fluorescence and white arrows indicate apoptotic cells. Each data point represents the mean** ± SEM of four replicates. Bars assigned different superscripts (a, b and c) are significantly different (*p* < 0.05). C, control diet; CS, control diet supplemented with 100 mg kg^−1^ silver nanoparticles (Ag-NPs); HC, high-carbohydrate (HC) diet; HCS, HC diet supplemented with 100 mg kg^−1^ Ag-NPs (the same below).

Hepatic apoptosis results of blunt snout bream subjected to different treatments are presented in Figure 2. Apoptotic cells are dyed with brown. In Figure 2A–D, we can see that there are more apoptotic cells (brown cells) in the CS group than in other groups. Apoptotic rates in the CS group were also significantly (*p* < 0.05) higher than those of other groups. In addition, the values of apoptotic rates of the HCS group were significantly (*p* < 0.05) higher than those of the C group, but there are no significant differences (*p* > 0.05) with the HC group.

### 3.5. Liver ATP and AMP Contents

**Figure 3:**
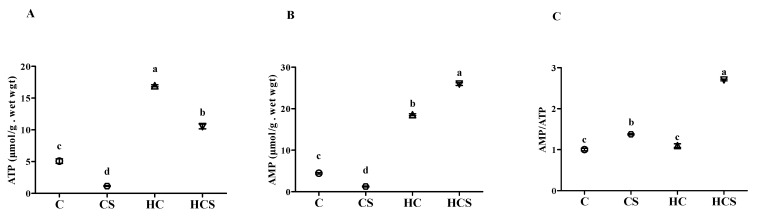
Hepatic ATP (**A**) and AMP (**B**) contents and the AMP/ATP ratio (**C**) of *M. amblycephala* subjected to different treatments. ATP, adenosine triphosphate; AMP, adenosine monophosphate. Each data point represents the mean ± SEM of four replicates. Bars assigned different superscripts (a, b, c and d) are significantly different (*p* < 0.05).

As can be seen from Figure 3, the lowest ATP and AMP contents were found in the CS group, while the highest values of AMP contents and AMP/ATP ratio were found in the HCS group. In addition, liver ATP and AMP contents of the HC group were significantly (*p* < 0.05) higher than those of the C group.

### 3.6. Protein Contents and Transcriptions of AMPKα

**Figure 4:**
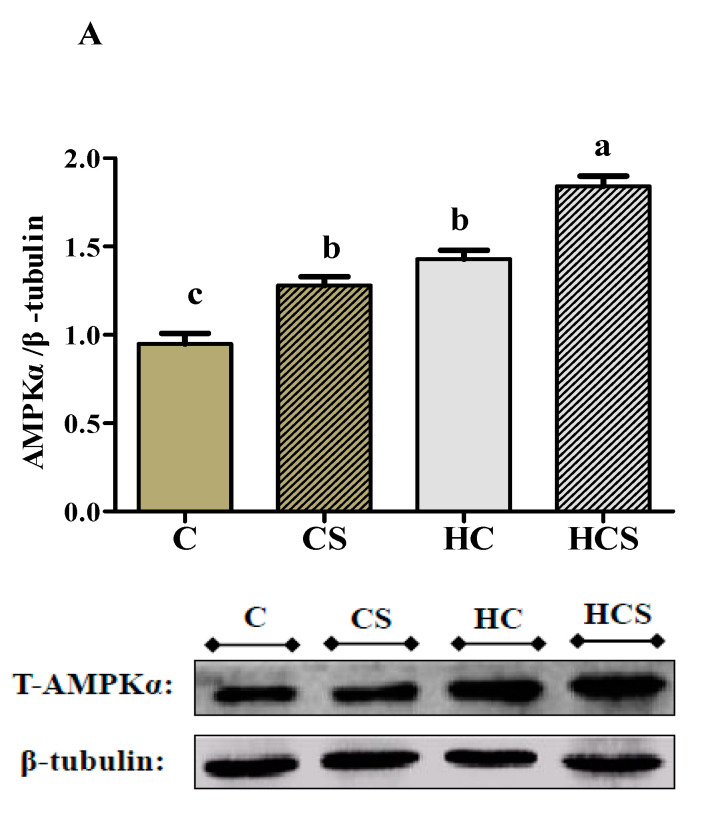

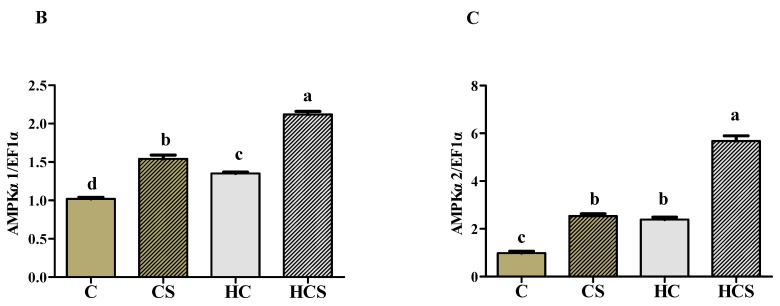
Hepatic content of T-AMPKα protein (**A**), transcriptions of AMPKα 1 (**B**), AMPKα 2 (**C**) *M. amblycephala* subjected to different treatments. Gels were loaded with 20 mg total protein per lane. Each data point represents the means ± SEM of four replicates. Bars assigned different superscripts (a, b, c and d) are significantly different (*p* < 0.05).

AMPKα protein contents, as well as AMPKα 1 and AMPKα 2 mRNA levels of the HCS group, were all significantly (*p* < 0.05) higher than those of other groups (Figure 4). In addition, AMPKα protein content and AMPKα 2 mRNA levels of the CS group were both significantly (*p* < 0.05) higher than those of the C group, but there were no significant differences (*p* > 0.05) with the HC group.

### 3.7. Transcriptions of Antioxidant-Related Genes

**Figure 5:**
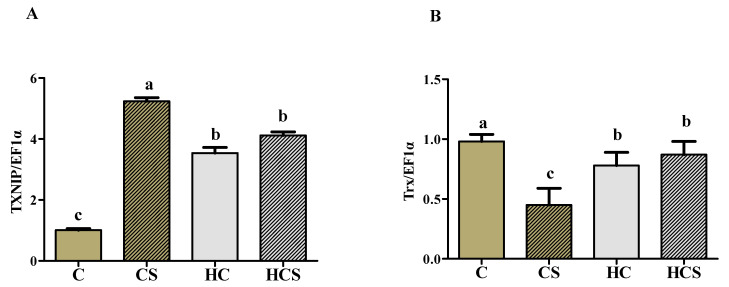

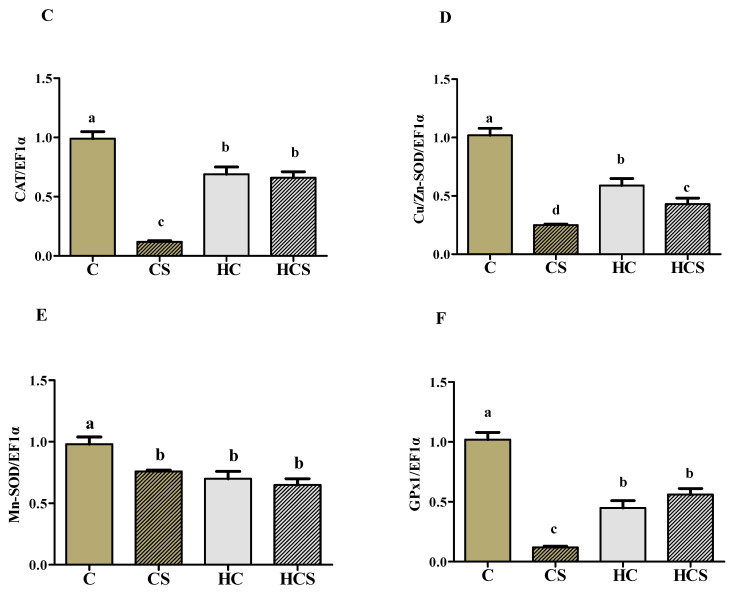
Hepatic transcriptional levels of antioxidant-related genes (**A**: TXNIP; **B**: Trx; **C**: CAT; **D**: Cu/Zn-SOD; **E**: Mn-SOD and **F**: GPx1) of *M. amblycephala* subjected to different treatments. Each data point represents the means ± SEM of four replicates. Bars assigned different superscripts (a, b, c and d) are significantly different (*p* < 0.05).

As can be seen from Figure 5, the transcriptions of Trx, CAT, Cu/Zn-SOD and GPx1 of the CS group were all significantly (*p* < 0.05) lower than those of other groups, while the opposite was true for TXNIP transcription. The transcriptions of Trx, CAT, Mn-SOD and GPx1 of the HCS group were all significantly (*p* < 0.05) lower than those of the C group, whereas the opposite was true for TXNIP. In addition, the values of TXNIP, Trx, CAT, Mn-SOD and GPx1 showed no significant differences (*p* > 0.05) between the HC and HCS groups.

### 3.8. Transcriptions of Inflammation-Related Genes

**Figure 6:**
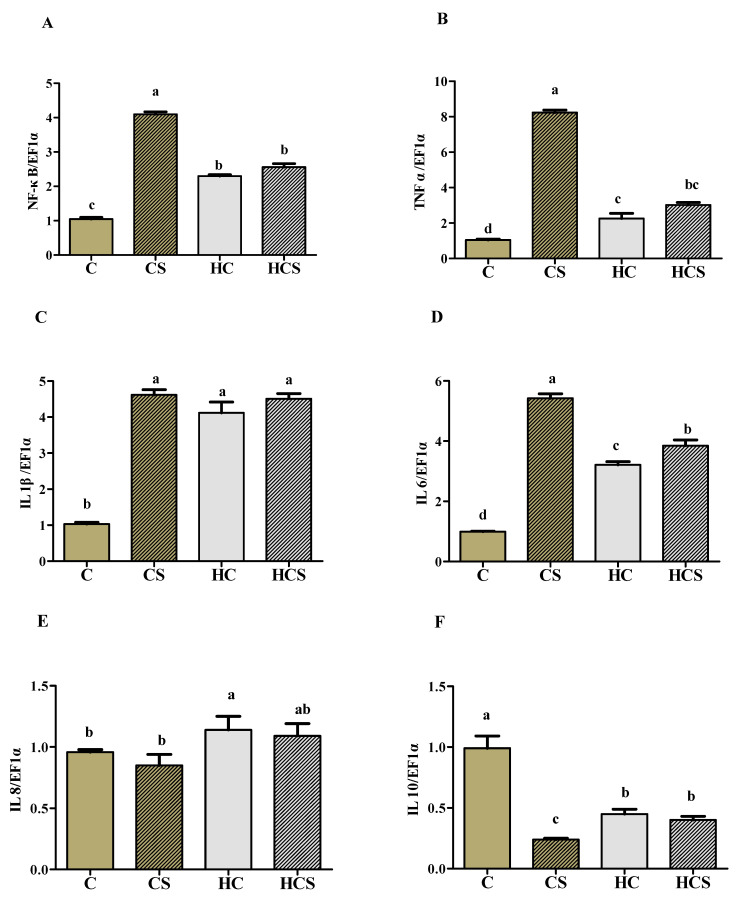
Hepatic transcriptional levels of inflammation-related genes (**A**: NF-κB; **B**: TNF α; **C**: IL 1β; **D**: IL 6; **E**: IL 8 and **F**: IL 10) of *M. amblycephala* subjected to different treatments. Each data point represents the means ± SEM of four replicates. Bars assigned different superscripts (a, b, c and d) are significantly different (*p* < 0.05).

As can be seen from Figure 6, the transcriptions of NF-κB, TNF α and IL 6 of the CS group were all significantly (*p* < 0.05) higher than those of other groups, while the opposite was true for IL 10 transcription. The transcriptions of NF-κB, TNF α, IL 1β and IL 8 of the HCS group were all significantly (*p* < 0.05) lower than those of the C group, whereas the opposite was true for IL 10. In addition, the values of NF-κB, TNF α, IL 1β, IL 8 and IL 10 showed no significant differences (*p* > 0.05) between the HC and HCS groups.

### 3.9. Transcriptions of Apoptosis-Related Genes and Protein

**Figure 7:**
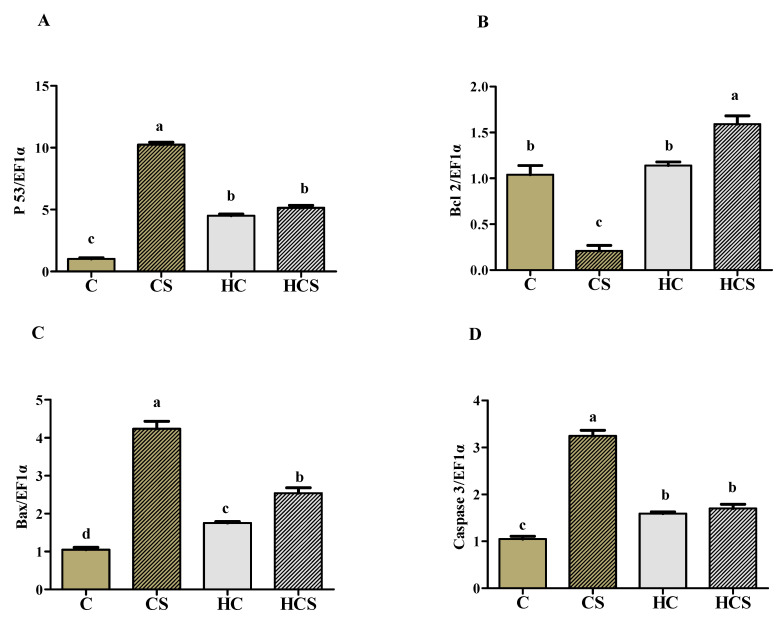

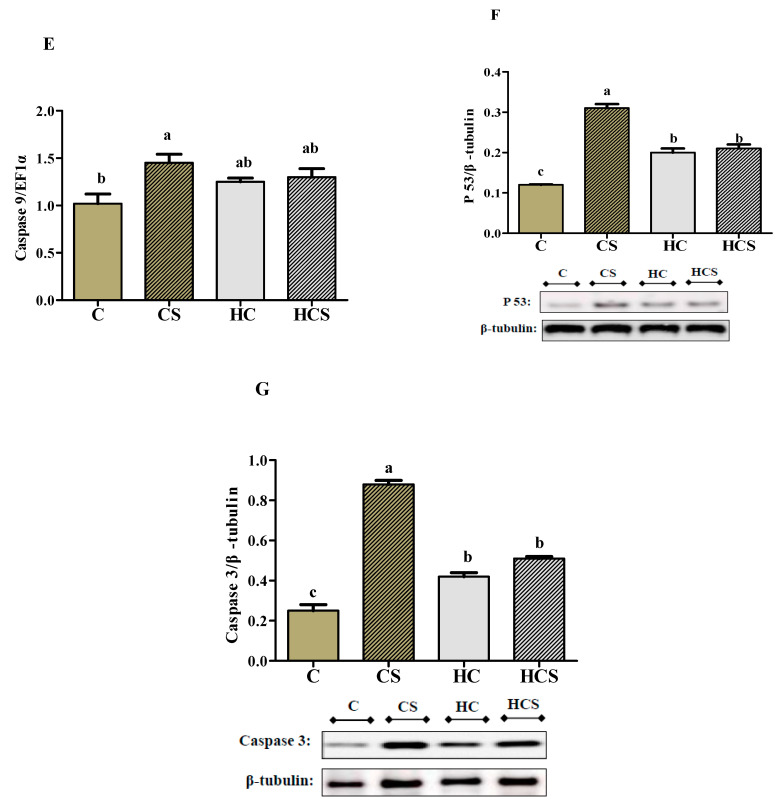
Hepatic transcriptional levels of apoptosis-related genes (**A**: P 53; **B**: Bcl 2; **C**: Bax; **D**: Caspase 3 and **E**: Caspase 9) and protein (**F**: P 53 and **G**: Caspase 3) of *M. amblycephala* subjected to different treatments. Gels were loaded with 20 mg total protein per lane. Each data point represents the means ± SEM of four replicates. Bars assigned different superscripts (a, b, c and d) are significantly different (*p* < 0.05).

As can be seen from Figure 7, P 53, Bax and caspase 3 mRNA levels, as well as P 53 and caspase 3 protein contents of the CS group, were all significantly (*p* < 0.05) higher than those of other groups, while the opposite was true for Bcl 2 transcription. P 53, caspase 3 and caspase 9 mRNA levels, as well as P 53 and caspase 3 protein contents of the HCS group, were all significantly (*p* < 0.05) higher than those of the C group, but there were no significant differences (*p* > 0.05) with the HC group. In addition, the transcriptions of Bcl 2 of the HCS group were significantly (*p* < 0.05) higher than those of other groups, but there were no significant differences (*p* > 0.05) between the C and HC groups.

## 4. Discussion

After the 12-week feeding trial, WGR, SGR, RFI and FCR of fish fed diets without Ag-NPs all tended to decrease with increasing dietary carbohydrate levels. These results might be due to the following facts: (a) carbohydrate-enriched diets can cause persistent hyperglycemia of fish, which might result in metabolic disorders, thus negatively affecting growth [31,32]; and (b) high-carbohydrate diets can reduce feed palatability and accelerate fish satiety, thus reducing feed consumption [33]. In addition, WGR, SGR and RFI were further remarkably decreased when fish were fed diets with Ag-NPs. Previous studies have demonstrated that AgNPs exposure could lead to serious pathology problems in fish tissues/organs, such as necrotic hepatocytes, hemocyte overfilling, hepatocyte enlargement and hepatocytes’ nuclear degeneration [2,34,35]. This may result in a decrease in growth performance of *M. amblycephala*. However, compared with the CS group, the WGR and SGR of the HCS group were remarkably higher, while the opposite was true for RFI and FCR. The results suggested that high-carbohydrate diets could improve growth performance of fish exposed to Ag-NPs. A reasonable explanation may be due to the increased ATP production of fish due to high-carbohydrate feeding, since it can accelerate the self-renewal of injured organisms [36], thereby alleviating Ag-NPs-induced negative effects on the growth of fish.

In this study, high dietary carbohydrate levels increased plasma activities of AST and ALT, levels of IL 1β and IL 6, liver contents of ROS and MDA, as well as the hepatocytes apoptosis rate than those of the C group, while the opposite was true for T-AOC, SOD and CAT. The results were in line with previous studies, in which the long-term intake of carbohydrate-enriched diets led to inflammation, hepatocytes apoptosis and low antioxidant ability in fish [37,38,39]. This was supported by the fact that: (a) pro-inflammatory cytokines containing IL 1β and IL 6 are increased when a host experiences inflammation [40]; and (b) the protective effects against oxidative damages could be directly reflected by the activities of some antioxidant enzymes, such as SOD and CAT, in fish, as they are in mammals [41]. In addition, dietary Ag-NPs supplementation further increased the values of AST, ALT, IL 1β, IL 6, ROS, MDA and hepatocyte apoptosis rate of fish fed C diets, but the opposite trend was true for SOD and CAT, suggesting increased inflammation and hepatocyte apoptosis, but decreased antioxidant capacity in the livers of *M. amblycephala* fed C diets. According to previous studies, Ag-NPs can react with the thiol groups of enzymes, including key components of the cell’s antioxidant defense mechanism, such as SOD and CAT [42,43]. As a consequence, Ag-NPs depleted the cell antioxidant defense mechanism, thereby resulting in a decrease in SOD and CAT activities and an increase in ROS levels. Then, overproduction of ROS could induce intracellular inflammatory stresses characterized by increased plasma IL 1β and IL 6 levels [44]. Meanwhile, multiple pro-inflammatory cytokines, such as IL 1β and IL 6, can trigger apoptosis, thereby stimulating programmed cell death [9,10]. It should be stated here that this information was mainly derived from mammals. The underlying mechanisms in fish still need further and more detailed studies. However, the values of AST, ALT, IL 1β, IL 6, ROS, MDA and hepatocyte apoptosis rate of the HCS group were all remarkably lower than those of the CS group, while the opposite was true for T-AOC, SOD and CAT. The results suggested that carbohydrate-enriched diets can enhance antioxidant capacity and inhibit inflammation and hepatocyte apoptosis of *M. amblycephala* fed with Ag-NPs. In order to characterize the corresponding mechanisms, molecular investigations were performed in a follow-up study.

AMPK is a stress-activated protein kinase that is activated in response to stresses that change the cellular AMP/ATP ratio, e.g., glucose load. In this study, hepatic ATP and AMP contents, AMPKα protein content and AMPKα 1 and AMPKα 2 mRNA levels all increased with increasing dietary carbohydrate levels. The most plausible explanation would be that high-carbohydrate diets could enhance glycolysis and glucose oxidation in the liver, thereby increasing ATP production [45]. Then, the increased ATP was hydrolyzed, thus leading to an increase in AMP content. In addition, the relatively high values of AMPKα protein content and AMPKα 1 and AMPKα 2 mRNA levels in the HC group were also not surprising; after an adaptation to carbohydrate-enhanced diets, AMPK may be activated to regulate glucose and lipid metabolism to maintain energy homeostasis [46]. As for Ag-NPs supplementation, it increased the values of the AMP/ATP ratio, AMPKα protein content and AMPKα 1 and AMPKα 2 mRNA levels, whereas the opposite was true for ATP content. This indicated that Ag-NPs could modify the intracellular energy state of fish. From these results, it is reasonable to suggest that Ag-NPs could cause mitochondrial dysfunction by inducing ROS generation, thereby resulting in a decrease in ATP content [47]. Meanwhile, the increased AMP/ATP ratio is considered as a positive signal, since it can activate AMPK, thereby enhancing the metabolism and immune function of fish [48].

Generally, activated AMPK can inhibit ROS production by accelerating TXNIP protein degradation [12], thereby regulating the antioxidant defense of organisms. However, such information in aquatic animals is extremely rare. In this study, hepatic transcriptions of Trx, CAT, Cu/Zn-SOD and GPx1 of fish fed HC diets were all significantly down-regulated compared to those in the C group, whereas the opposite was true for TXNIP. These results were in line with those related to antioxidant enzyme activities, suggesting that HC diets can reduce the hepatic antioxidant capacity of fish. Previous studies have shown that the activation of TXNIP by high glucose could inhibit Trx activity, thereby decreasing the antioxidant capacity of organisms [49,50], as might be reflected by decreasing the activity and expression of endogenous antioxidant enzymes such as SOD and GPx1. As for Ag-NPs supplementation, hepatic transcriptions of Trx, CAT, Cu/Zn-SOD, Mn-SOD and GPx1 in the CS group were all remarkably lower than those of other groups, whereas the opposite was true for TXNIP. These results might be due to the fact that the overproduction of ROS induced by Ag-NPs could activate TXNIP, thereby inhibiting the function of Trx as a major cellular redox regulator [51]. This may decrease the activity and expression of Cu/Zn-SOD, Mn-SOD and GPx1, thus resulting in an increase in oxidative stress of fish. However, the aforementioned studies are mainly focused on mammals. The exact mechanisms in fish still warrant further in-depth studies. In addition, the values of Trx, CAT, Cu/Zn-SOD and GPx1 were increased in the HCS group, suggesting the intake of an HC diet could enhance the antioxidant capacity of this fish when faced with Ag-NPs. This was supported by the fact that the activation of AMPK could significantly up-regulate the transcriptional activities of Trx by accelerating TXNIP protein degradation, thus promoting cellular defense against oxidative stress [12,49].

In addition, activated AMPK can also suppress inflammation via the inhibition of the NF-κB pathway [13]. In this study, hepatic NF-κB, TNF α, IL 1β and IL 6 expression in fish fed diets without Ag-NPs were all significantly increased with increasing dietary carbohydrate levels, whereas the opposite was true for IL 10 expression. This indicated that high-carbohydrate diets caused inflammation in the liver of *M. amblycephala*. This was supported by the fact that IL 1β and IL 6 are essential pro-inflammatory cytokines, and their secretion can lead to a pro-inflammatory cascade, including production of TNFα, thereby triggering inflammation [52]. Meanwhile, the decreased expression of IL 10 was also considered to be a positive signal for inflammation, since IL 10 could inhibit the synthesis of pro-inflammatory cytokines [53]. Furthermore, Ag-NPs supplementation further significantly up-regulated expression of NF-κB, TNF α, IL 1β and IL 6 of fish fed with C diets. Previous studies have demonstrated that Ag-NPs could stimulate ROS generation, thereby activating NF-κB-mediated pathways, which lead to pro-inflammatory genes (TNF α, IL 1β and IL 6) expression [54,55]. However, the expression of NF-κB and pro-inflammatory genes of the HCS group were all remarkably lower than those of the CS group, which was in line with plasma IL 1β and IL 6 levels. This may be due to the fact that activated AMPK can deacetylate P 65 protein (an activator of NF-κB signaling), thus inhibiting NF-κB mediated pro-inflammatory pathways [56,57]. Moreover, activated AMPK can also increase the expression of anti-inflammatory cytokines (such as IL 10), thus decreasing inflammatory stress of tissues [58]. However, this is also the case in mammals. Whether fish show a similar mechanism is still uncertain, and this warrants further study. In addition, the expressions of NF-κB, TNF α, IL 1β and IL 6 of the HCS group were all generally higher than those of the HC group. This may be explained by the fact that growth performance is decreased in the HCS group. 

Furthermore, the underlying molecular mechanism of hepatocyte apoptosis has been investigated in this study. Hepatic protein contents of P 53 and caspase 3, as well as transcriptions of P 53, Bax and caspase 3, in fish fed HC diets were all significantly increased than those of the C group, suggesting carbohydrate-enriched diets induce apoptosis in the liver of *M*. *amblycephala*. According to previous studies, the activation of P 53 by high glucose could up-regulate expression of its downstream pro-apoptotic genes such as Bax and caspase 3 [10,59]. Then, activated caspase 3 can cleave cellular substrates, ultimately leading to cell apoptosis and death [60]. As for Ag-NPs supplementation, hepatic transcriptions of P 53, Bax, caspase 3 and caspase 9 of the CS group were all higher than in other groups. This might be due to the fact that Ag-NPs can activate P 53 by increasing its phosphorylation at Ser-15, thereby accelerating P 53-mediated apoptosis [61]. However, the expressions of P 53 and pro-apoptotic genes of the HCS group were all remarkably lower than those of the CS group, whereas the opposite was true for an anti-apoptotic gene (Bcl 2). This indicated that HC diets could inhibit apoptosis in the liver of *M*. *amblycephala* facing Ag-NPs, which correlates with the result of the hepatocyte apoptosis rate. A previous study has shown that, under high glucose, AMPK activation deacetylates P 53 protein to increase its degradation, thereby reducing cell apoptosis [62]. This may also exist in fish as it does in mammals. Interestingly, in response to extreme glucose deprivation, AMPK activation could induce phosphorylation of P 53 protein at Ser-15, resulting in P 53 activation and stabilization [63,64]. This may further demonstrate that high dietary carbohydrate levels are beneficial for *M*. *amblycephala* facing Ag-NPs. It should be stated here that this information was mainly derived from mammals. The underlying mechanisms in fish still require further and more detailed studies.

## 5. Conclusions

In summary, the results obtained in this study suggest that a high-carbohydrate diet can attenuate Ag-NPs-induced hepatic oxidative stress, inflammation and apoptosis of *M*. *amblycephala* via the activation of AMPK and the enhancement of antioxidant enzyme activities, coupled with the down-regulated transcriptions of NF-κB-mediated pro-inflammatory cytokines and P 53-mediated pro-apoptotic genes.

## Figures and Tables

**Table 1:** Formulation and proximate composition of the experimental diets.

	Control Diet	High-Carbohydrate Diet
***Formulation*** **(*%*)**		
Fish meal	5.00	5.00
Soybean meal	30.00	30.00
Rapeseed meal	18.40	18.40
Cottonseed meal	15.00	15.00
Soybean oil	3.60	3.60
Corn starch	12.00	25.00
Microcrystalline cellulose	13.00	0.00
Calcium biphosphate	1.80	1.80
Premix *	1.20	1.20
***Proximate composition*** **(*% air-dry basis*)**		
Moisture	8.79	8.96
Crude lipid	4.97	5.25
Ash	7.00	6.96
Crude protein	31.71	31.94
Crude fiber	15.76	3.00
Nitrogen-free extract ^†^	31.77	43.89
Energy (MJ/kg)	18.89	18.82

* Premix supplied the following minerals and/or vitamins (per kg of premix): CuSO_4_·5H_2_O, 2.0 g; FeSO_4_·7H_2_O, 25 g; ZnSO_4_·7H_2_O, 22 g; MnSO_4_·4H_2_O, 7 g; Na_2_SeO_3_, 0.04g; KI, 0.026 g; CoCl_2_·6H_2_O, 0.1 g; vitamin A, 900,000 IU; vitamin D, 200,000 IU; vitamin E, 4500 mg; vitamin K_3_, 220 mg; vitamin B_1_, 320 mg; vitamin B_2_, 1090 mg; vitamin B_5_, 2000 mg; itamin B_6_, 500 mg; vitamin B_12_, 1.6 mg; vitamin C, 5000 mg; pantothenate, 1000 mg; folic acid, 165 mg; choline, 60,000 mg.^†^ Calculated by difference (100-moisture-crude protein-crude lipid-ash-crude fiber).

**Table 2:** Nucleotide sequences of the primers used to assay gene expressions by real-time PCR.

Target Gene	Forward (5′-3)	Reverse (5′-3)	Accession Numbers or References
AMPKα 1	AGTTGGACGAGAAGGAG	AGGGCATACAAAATCAC	ARF07712.1
AMPKα 2	ACAGCCCTAAGGCACGATG	TGGGTCGGGTAGTGTTGAG	KX061841
TXNIP	CAGACTTGCTGTCCCCTAC	CTCCAGAACCAACTTATCG	MW582526
Trx	TCACAATCGCCTTCAATC	CTCCCTTCTTACCCACAA	[29]
CAT	CAGTGCTCCTGATACCCAGC	TTCTGACACAGACGCTCTCG	[29]
Cu/Zn-SOD	AGTTGCCATGTGCACTTTTCT	AGGTGCTAGTCGAGTGTTAGG	KF479046.1
Mn-SOD	AGCTGCACCACAGCAAGCAC	TCCTCCACCATTCGGTGACA	KF195932.1
GPx1	GAACGCCCACCCTCTGTTTG	GAACGCCCACCCTCTGTTTG	KF378713.1
NF-κB	GAAGAAGGATGTGGGAGATG	TGTTGTCGTAGATGGGCTGAG	[30]
TNFα	TGGAGAGTGAACCAGGACCA	AGAGACCTGGCTGTAGACGA	KU976426.1
IL 1β	ACGATAAGACCAGCACGACC	CTGTTTCCGTCTCTCAGCGT	[30]
IL 6	CAGCAGAATGGGGGAGTTATC	CTCGCAGAGTCTTGACATCCTT	KJ755058.1
IL 8	CAGAGAGTCGACGCATTGGT	ATTCACGGTGCTTTGTTGGC	[31]
IL 10	GTGTTTTCGGGTGCAAGTGG	ATGAACGAGATCCTGCGCTT	[31]
P 53	CAGCAGGAGCCAATCCATCA	ACGTACTCCCCAGACCTGAA	[31]
Bcl 2	CCAACTCATCAGGAAACAA	GGGTGCTGCGGGTAAC	EU490408.1
Bax	ATCCAGCCAGCATCGT	CACTATCCCCAAGACCC	AF231015.1
Caspase 3	TCGTTCGTCTGTGTCCTGTTGAG	GCTGTGGAGAAGGCGTAGAGG	KY006115.1
Caspase 9	AATAAAGCACCGAGCG	GGGAGGAGGCCGATGAGCACTATCT	KM604705.1
EF1α	CTTCTCAGGCTGACTGTGC	CCGCTAGCATTACCCTCC	X77689.1

AMPKα 1, AMP-activated protein kinase α 1; AMPKα 2, AMP-activated protein kinase α 2; TXNIP, thioredoxin-interacting protein; Trx, thioredoxin; CAT, catalase; Cu/Zn-SOD, copper/zinc superoxide dismutase; Mn-SOD, manganese superoxide dismutase; GPx1, glutathione peroxidase 1; NF-κB, nuclear factor kappa B; TNF α, tumor necrosis factor α; IL 1β, interleukin 1β; IL 6, interleukin 6; IL 8, interleukin 8; IL 10, interleukin 10; EF1α, elongation factor 1α.

**Table 3:** Growth performance and feed utilization of blunt snout bream subjected to different treatments.

Parameters	Groups				*p*-Value
	C	CS	HC	HCS	
Initial weight (g)	20.12 ± 0.07	20.42 ± 0.23	20.41 ± 0.21	20.22 ± 0.12	>0.05
Final weight (g)	124.57 ± 5.18 ^a^	65.69 ± 8.41 ^d^	101.24 ± 4.11 ^b^	88.84 ± 4.31 ^c^	<0.05
WGR † (%)	462.98 ± 10.20 ^a^	225.12 ± 9.57 ^d^	394.19 ± 8.31 ^b^	336.12 ± 5.61 ^c^	<0.05
SGR§(% day^−1^)	3.12 ± 0.02 ^a^	1.95 ± 0.01 ^d^	2.81 ± 0.04 ^b^	2.64 ± 0.01 ^c^	<0.05
RFI || (% body weight d^−1^)	3.35 ± 0.01	2.94 ± 0.03	3.12 ± 0.01	2.81 ± 0.03	>0.05
FCR	1.35 ± 0.02	1.58 ± 0.01	1.30 ± 0.02	1.27 ± 0.02	>0.05

C, control diet; CS, control diet supplemented with 100 mg kg^−1^ silver nanoparticles (Ag-NPs); HC, high-carbohydrate (HC) diet; HCS, HC diet supplemented with 100 mg kg^−1^ Ag-NPs (the same below).† Weight gain rate (WGR, %) = (W_t_ − W_0_) × 100/W_0_.§Specific growth rate (SGR)= (LnW_t_ − LnW_0_) × 100/T, where W_0_ and W_t_ are the initial and final body weights, and T is the culture period in days.|| Relative feed intake (RFI) = Feed intake (g) × 100/[(initial fish weight (g) + final fish weight (g) + dead fish weight (g)) × days reared/2]. Feed conversion ratio (FCR) = feed consumption (g)/fish weight gain (g). Values are means ± S.E.M. of four replications. Means in the same line with different superscripts (a, b, c and d) are significantly different (*p* < 0.05).

**Table 4:** Plasma and liver parameters of blunt snout bream subjected to different treatments.

Parameters	Groups				*p*-Value
	C	CS	HC	HCS	
***Plasma parameters***					
AST (U/L)	23.47 ± 0.12 ^c^	43.11 ± 0.11 ^a^	33.10 ± 0.21 ^b^	35.47 ± 0.35 ^b^	<0.05
ALT (U/L)	2.01 ± 0.01 ^d^	6.24 ± 0.03 ^a^	3.45 ± 0.01 ^c^	4.85 ± 0.14 ^b^	<0.05
IL 1β (ng/L)	1.21 ± 0.01 ^d^	7.11 ± 0.02 ^a^	2.31 ± 0.01 ^c^	5.34 ± 0.03 ^b^	<0.05
IL 6 (ng/L)	21.24 ± 0.31 ^c^	45.72 ± 0.65 ^a^	33.54 ± 1.04 ^b^	34.78 ± 0.52 ^b^	<0.05
***Liver parameters***					
ROS (% control)	1.02 ± 0.02 ^c^	7.14 ± 0.01 ^a^	4.25 ± 0.01 ^b^	4.64 ± 0.03 ^b^	<0.05
T-AOC (U/mg protein)	1.45 ± 0.01 ^a^	0.35 ± 0.01 ^c^	0.54 ± 0.02 ^b^	0.55 ± 0.01 ^b^	<0.05
SOD (U/mg protein)	12.36 ± 0.04 ^a^	6.35 ± 0.04 ^c^	10.19 ± 0.11 ^b^	9.22 ± 0.05 ^b^	<0.05
CAT (U/mg protein)	20.21 ± 0.22 ^a^	4.49 ± 0.03 ^c^	11.34 ± 0.21 ^b^	10.18 ± 0.02 ^b^	<0.05
MDA (nmol/mg protein)	2.34 ± 0.02 ^c^	10.29 ± 0.12 ^a^	8.21 ± 0.01 ^b^	8.61 ± 0.05 ^b^	<0.05

AST, alanine transaminase; ALT, aspartate aminotransferase; IL 1β, interleukin 1β; IL 6, interleukin 6; ROS, reactive oxygen species; T-AOC, total anti-oxidation capacity; SOD, superoxide dismutase; CAT, catalase; MDA, malondialdehyde. Values are means ± S.E.M. of four replications. Means in the same line with different superscripts (a, b, c and d) are significantly different (*p* < 0.05).

## Data Availability

Data is contained within the article.
